# Ca_v_1.3 (*CACNA1D*) L‐type Ca^2+^ channel dysfunction in CNS disorders

**DOI:** 10.1113/JP270672

**Published:** 2016-02-29

**Authors:** Alexandra Pinggera, Jörg Striessnig

**Affiliations:** ^1^ Department of Pharmacology and ToxicologyCenter for Molecular BiosciencesUniversity of InnsbruckInnrain 80/826020InnsbruckAustria

## Abstract

Ca_v_1.3 belongs to the family of voltage‐gated L‐type Ca^2+^ channels and is encoded by the *CACNA1D* gene. Ca_v_1.3 channels are not only essential for cardiac pacemaking, hearing and hormone secretion but are also expressed postsynaptically in neurons, where they shape neuronal firing and plasticity. Recent findings provide evidence that human mutations in the *CACNA1D* gene can confer risk for the development of neuropsychiatric disease and perhaps also epilepsy. Loss of Ca_v_1.3 function, as shown in knock‐out mouse models and by human mutations, does not result in neuropsychiatric or neurological disease symptoms, whereas their acute selective pharmacological activation results in a depressive‐like behaviour in mice. Therefore it is likely that *CACNA1D* mutations enhancing activity may be disease relevant also in humans. Indeed, whole exome sequencing studies, originally prompted to identify mutations in primary aldosteronism, revealed *de novo CACNA1D* missense mutations permitting enhanced Ca^2+^ signalling through Ca_v_1.3. Remarkably, apart from primary aldosteronism, heterozygous carriers of these mutations also showed seizures and neurological abnormalities. Different missense mutations with very similar gain‐of‐function properties were recently reported in patients with autism spectrum disorders (ASD). These data strongly suggest that *CACNA1D* mutations enhancing Ca_v_1.3 activity confer a strong risk for – or even cause – CNS disorders, such as ASD.

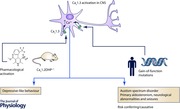

**Abbreviations**
ASD: autism spectrum disorders
APA: aldosterone producing adenoma
CaM: calmodulin
ExAC: Exome Aggregation Consortium
PASNA: primary aldosteronism, seizures and neurological abnormalities
SNPs: single nucleotide polymorphisms
Q_ON_: ON‐gating charge movement

## Introduction

L‐type channels are one of three classes of voltage‐gated Ca^2+^ channels characterized by high sensitivity to channel blockers (Ca^2+^ antagonists), such as dihydropyridines. Among the four L‐type channel isoforms (Ca_v_1.1–1.4), Ca_v_1.2 and Ca_v_1.3 are expressed in most electrically excitable cells in mammals, including the brain (Koschak *et al*. 2014; Striessnig *et al*. 2014, 2015; Vandael *et al*. 2015, for recent reviews). Ca^2+^ entry through these isoforms supports physiological functions that are mediated by electrical activity, including neurotransmitter release in sensory cells, control of neuronal excitability and plasticity, hormone secretion, heart rhythm and contractility as well as smooth muscle contraction (Hofmann *et al*. 2014; Koschak *et al*. 2014; Striessnig *et al*. 2014, 2015; Vandael *et al*. 2015; Zamponi *et al*. 2015, for recent reviews). However, due to differences in their biophysical properties, protein interactions, expression patterns and modulation by other signalling pathways, they contribute to these physiological functions in different ways (Striessnig *et al*. 2014; Zamponi *et al*. 2015).

Insight into the physiological functions of Ca_v_1.3 has mainly been obtained from Ca_v_1.3‐deficient and mutant mice (Platzer *et al*. 2000; Sinnegger‐Brauns *et al*. 2004) and from homozygous loss‐of‐function mutations in humans (Baig *et al*. 2011). The essential role of Ca_v_1.3 for different organ functions has recently been extensively reviewed (Koschak *et al*. 2014; Striessnig *et al*. 2014; Zamponi *et al*. 2015) and is therefore not discussed in detail here. In the past few years, the discovery of human gain‐of‐function mutations in *CACNA1D*, encoding the pore‐forming Ca_v_1.3 α_1_‐subunit, revealed novel physiological and pathophysiological functions of this channel (Azizan *et al*. 2013; Scholl *et al*. 2013). Gain‐of‐function mutations in Ca_v_1.2 (*CACNA1C*) have already been reported in 2004 as the cause for Timothy syndrome (Splawski *et al*. 2004). Ca_v_1.2 is expressed at higher abundance than Ca_v_1.3 in the brain and is the major L‐type channel isoform in the heart and vascular smooth muscle (Striessnig *et al*. 2014; Zamponi *et al*. 2015). Timothy syndrome patients exhibit cardiovascular (long‐QT syndrome) as well as developmental abnormalities (facial abnormalities, syndactyly), and a large percentage of the affected individuals also show autism, mental retardation and seizures (Splawski *et al*. 2004, 2005). This was the first indication that L‐type Ca^2+^ channels could contribute to neuropsychiatric and neurological disease. As summarized in recent reviews (Bhat *et al*. 2012; Smoller *et al*. 2013; Zamponi *et al*. 2015) substantial evidence has accumulated since then for a prominent role of *CACNA1C* single nucleotide polymorphisms (SNPs) for neuropsychiatric disease risk. In addition also auxiliary α_2_δ and β subunits as well as other Ca_v_ α_1_‐subunits have been implicated in autism spectrum disorders (ASD) (for review Breitenkamp *et al*. 2015; Soldatov, 2015).

Here we discuss recent evidence from human genetics, strongly pointing to an important role of Ca_v_1.3 dysfunction in psychiatric disorders. As Ca_v_1.3 channels are also expressed in many tissues outside the brain, *CACNA1D* missense mutations causing a gain‐of‐function are likely to be associated with other (syndromic) organ dysfunctions. Indeed, activating Ca_v_1.3 missense mutations associated with CNS disorders were first discovered in whole exome sequencing studies identifying a causal role of *CACNA1D* mutations for primary aldosteronism. We will therefore discuss risk mutations for CNS disorders in the context of mutations causing primary aldosteronism.

## Ca_v_1.3 dysfunction in primary aldosteronism and CNS disease

The first disorders caused by mutations permitting enhanced Ca_v_1.3 Ca^2+^ channel activity were discovered as somatic *CACNA1D* missense mutations in, typically benign, aldosterone‐producing adrenal adenomas (APAs) in patients with primary aldosteronism (Azizan *et al*. 2013; Scholl *et al*. 2013). APAs are found in about 5% of individuals referred to hypertension clinics. *CACNA1D* mutations were not the first somatic mutations to be described in APAs responsible for excess aldosterone production in these tumours. Also mutations in genes for the K^+^‐channel, *KCNJ5*, the Na^+^/K^+^‐ATPase, *ATP1A1*, and the Ca^2+^‐ATPase, *ATP2B3*, were identified and all mutation‐induced functional changes could be predicted to enhance intracellular Ca^2+^ signalling. For example, in the case of *ATP1A1* mutations this is due to impaired Na^+^/K^+^‐ATPase activity which triggers depolarization‐induced Ca^2+^ entry through voltage‐dependent Ca^2+^ channels (Azizan *et al*. 2013; Beuschlein *et al*. 2013; Scholl *et al*. 2013).

Somatic mutations in *CACNA1D* and the other genes are not limited to APAs but are also found in the more frequent multinodular adrenals and in diffuse hyperplasia (Dekkers *et al*. 2014; Fernandes‐Rosa *et al*. 2015; Scholl *et al*. 2015 *a*). Only one nodule, usually the aldosterone synthase‐positive one, harbours a mutation in multinodular glands suggesting that the mutations are causative in aldosterone hypersecretion but not in nodule formation (Dekkers *et al*. 2014; Fernandes‐Rosa *et al*. 2015; Scholl *et al*. 2015 *a*).

**Figure 1:**
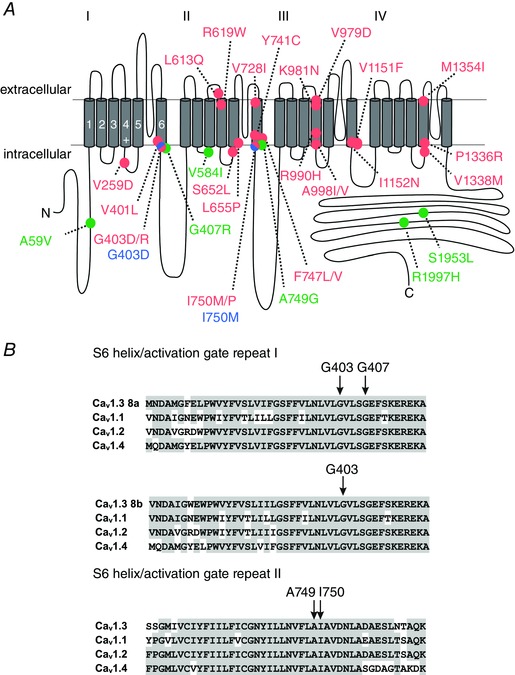
**Somatic and germline *CACNA1D* mutations reported in human disease** *A*, *CACNA1D* missense mutations are illustrated by circles. Somatic missense mutations identified in APAs are highlighted in red (Azizan *et al*. 2013; Scholl *et al*. 2013; Fernandes‐Rosa *et al*. 2014; Akerstrom *et al*. 2015; Nishimoto *et al*. 2015; Wang *et al*. 2015; Scholl *et al*. 2015 *a*). So far, only mutations V259D, G403D/R, I750M and P1336R have been functionally characterized, and they show strong gain‐of‐function phenotypes (Azizan *et al*. 2013; Scholl *et al*. 2013). G403R and I750M were also identified as *de novo* germline mutations in two patients with primary aldosteronism, seizures and neurological abnormalities (PASNA), highlighted in blue (Scholl *et al*. 2013). Mutations identified in individuals with ASD are shown in green. Mutations G407R (exon 8a), A749G and V584I were identified in the affected individuals but absent in parents or unaffected siblings and therefore classified as *de novo* (Iossifov *et al*. 2012; O'Roak *et al*. 2012; De Rubeis *et al*. 2014). Mutations A59V, S1953L and R1997H were detected in a case‐control sample (De Rubeis *et al*. 2014). Mutations G407R and A749G have been characterized and show a pronounced gain‐of‐function (Fig. 2, Pinggera *et al*. 2015). *B*, alignment of amino acid sequence of transmembrane S6‐helices of repeats I and II containing the activation gates at their cytoplasmic ends. They are highly conserved among α_1_ subunits of the L‐type Ca^2+^ channel family. Ca_v_1.3 undergoes alternative splicing in the activation gate of repeat I (exons 8a *vs*. 8b) (Baig *et al*. 2011). Positions mutated in ASD and PASNA are located in close proximity to each other as indicated in the alignment. Whereas ASD‐linked mutation G407R only occurs in exon 8a (Pinggera *et al*. 2015), G403D was identified in both exons in APAs but was present in exon 8b in PASNA (Scholl *et al*. 2013). (Alignment was performed by ClustalW using the following reference sequences: Ca_v_1.3 EU363339, Ca_v_1.1 Q02789, Ca_v_1.2 NP_001153005.1, Ca_v_1.4 Q9JIS7; conserved residues are highlighted in grey.)

Although T‐type (Ca_v_3 family, Perez‐Reyes, 2003; Zamponi *et al*. 2015 for review) and L‐type channels were known to be present in zona glomerulosa cells (Hunyady *et al*. 1994; Rossier *et al*. 1996; Hu *et al*. 2012), activating mutations in *CACNA1D* and, more recently, also in *CACNA1H* (Ca_v_3.2 T‐type channel, Scholl *et al*. 2015 *b*) outed these channels as the most relevant ones in human aldosterone producing cells. Since Ca^2+^ is the critical second messenger responsible for aldosterone production (for details see Barrett *et al*. in this series) it was likely that the *CACNA1D* missense mutations induce changes in either channel gating or channel expression that lead to enhanced Ca^2+^ entry during electrical activity of a zona glomerulosa cell. So far, at least 24 mutations in 20 different positions have been described in APAs (Fig. 1). Four of these mutations have been experimentally characterized and revealed functional changes compatible with a gain‐of‐function phenotype (Azizan *et al*. 2013; Scholl *et al*. 2013). This involved pronounced slowing of current inactivation during depolarizing stimuli, activation of channel current at more negative voltages and higher single channel open probability. For mutation I750M this will be discussed in more detail below.

Altogether these findings clearly demonstrate that the gating changes induced by the APA mutations result in enhanced Ca^2+^ signalling through Ca_v_1.3 channels in these tumour cells. It is likely that increased Ca^2+^ entry occurs during T‐type (Ca_v_3.2) Ca^2+^ channel‐sustained membrane oscillations (Hu *et al*. 2012). Moreover, this raises the important question whether such mutations could also contribute to the risk for neurological or neuropsychiatric disorders.

Ca_v_1.3 channels are widely expressed in the CNS (Striessnig *et al*. 2014; Zamponi *et al*. 2015 for review) and in neurons with different electrical activity patterns. They affect neuronal activity in a complex manner. Through coupling to Ca^2+^‐activated K^+^ channels they contribute to spike‐frequency adaptation and post‐burst afterhyperpolarization (McKinney *et al*. 2009), thus decreasing excitability. Conversely, these channels activate at relatively low (subthreshold) voltages, which allows them to create dendritic plateau potentials, during which neurons can fire tonically (Olson *et al*. 2005). Moreover, Ca_v_1.3 signalling is required for normal neuronal development, synapse maturation as well as synaptic pruning (Day *et al*. 2006; Hirtz *et al*. 2011, 2012). Ca_v_1.3 deficiency in mice leads to a relatively benign CNS/sensory phenotype. This includes deafness (due to inner hair cell dysfunction), reduced drug‐taking (for details see article by Kabir *et al*. in this issue) and antidepressant‐like behaviour (for recent review see Striessnig *et al*. 2014; Zamponi *et al*. 2015). In contrast, acute selective pharmacological activation of Ca_v_1.3 channels *in vivo* in mice (Sinnegger‐Brauns *et al*. 2004; Hetzenauer *et al*. 2006) induces a depression‐like phenotype and neuronal activation in a specific set of mainly limbic, hypothalamic and brainstem areas, which are associated with integration of emotion‐related behaviours (Hetzenauer *et al*. 2006). These experiments were performed in mice expressing dihydropyridine‐insensitive Ca_v_1.2 channels (Ca_v_1.2DHP^−/−^). Application of the Ca^2+^ channel activator BayK8644 therefore allowed the selective activation of Ca_v_1.3 channels in the brain (Sinnegger‐Brauns *et al*. 2004). These animal studies suggest that Ca_v_1.3 gain‐of‐function mutations could also increase risk for CNS abnormalities in humans presenting as neuropsychiatric disorders or epilepsy. Indeed, evidence that dysfunctional Ca_v_1.3 channels with gating properties resembling those found in APA mutations (and thus capable of enhancing Ca^2+^ currents through Ca_v_1.3 channels) provide a strong risk for CNS disease came from two patients with germline APA mutations (Scholl *et al*. 2013).

## *CACNA1D* mutations associated with primary aldosteronism with seizures and neurological abnormalities

In the course of characterizing somatic *CACNA1D* APA mutations, Scholl *et al*. (2013) discovered two mutations identified in APAs as germline *de novo* mutations in two patients with primary aldosteronism seizures and neurological abnormalities, a syndrome which was termed PASNA.

Patient 1 carried a copy of the G403D mutation (in alternative exon 8b) and was a 3‐year old female of European ancestry with no significant family history (Scholl *et al*. 2013). After birth she developed sinus bradycardia (day 1), and was successfully treated for transient hypoglycaemia (day 2), respiratory distress (day 3), atrioventricular block with prolonged QT‐interval and elevated blood pressure. Echocardiograms demonstrated biventricular hypertrophy, a ventricular septal defect and a patent foramen ovale with mild persistent pulmonary hypertension of the newborn. Primary aldosteronism (elevated aldosterone, low plasma renin activity) and hypokalaemia were diagnosed at age 1 month. Treatment with amlodipine normalized blood pressure, and resolved biventricular hypertrophy. Brain imaging at age 1 month was suggestive of periventricular leukomalacia, two regions with previous haemorrhages (right occipital region and left periventricular parenchyma) and ventriculomegaly. Further development was characterized by failure to thrive and a global developmental delay. Cardiac abnormalities resolved and no evidence for gastrointestinal disease was obtained. The patient developed epilepsy and was diagnosed with cortical blindness, spasticity and cerebral palsy. The first generalized tonic‐clonic seizure (month 7) was not associated with brain CT changes and there was no history of febrile illness or evidence for serum electrolyte abnormalities. Despite pharmacotherapy one generalized tonic‐clonic seizure per month developed until age 12 months, when seizure frequency increased. EEG showed recurrent spikes emanating predominantly from the right temporoparietal occipital region, and to a lesser extent, independently, from the left temporal region without a clinical correlate. At 3 years she was not ambulatory and not verbal (global developmental delay). The blood pressure and serum K^+^ levels were normal; recent medications included rufinamide, valproic acid, ranitidine, baclofen and levetiracetam (Scholl *et al*. 2013).

Patient 2 carried a copy of the I750M mutation and was a 10‐year old African American female with no significant family history (Scholl *et al*. 2013). Delivery was by Caesarean section at 41 weeks gestational age after uterine rupture. She required resuscitation and was subsequently diagnosed with cerebral palsy, spastic quadriplegia and mild athetosis, severe generalized intellectual disability, complex partial seizures of likely right hemispheric origin, generalized seizures, a movement disorder with verbal outbursts, and a sleep disorder. Treatment included oxcarbazepine, risperidone and later levetiracetam. Brain MRI was normal. The patient developed hypertension before the age of 5 years and at the age of 8 years also significant hypokalaemia. In addition, high aldosterone levels were found despite suppressed plasma renin activity. Abdominal CT scan at the age of 9 years showed normal appearance of the adrenal glands. Mild left ventricular hypertrophy was noted on echocardiogram. Hypertension was treated with clonidine, and later spironolactone (Scholl *et al*. 2013).

There was no family history of early‐onset hypertension or seizures in these patients. A common finding is the presence of hyperaldosteronism (with hypertension and hypokalaemia), seizures and generalized intellectual disability and spasticity. Hyperaldosteronism is a symptom expected from these mutations. The presence of seizures in both patients suggests that Ca_v_1.3 gain‐of‐function may also contribute to epilepsy risk.

Based on the published medical histories, it cannot be excluded that severe perinatal complications (asphyxia in patient 1, resuscitation in patient 2) caused the epilepsy in both patients. However, this neurological phenotype also prompted us to screen the literature for additional *CACNA1D* variants reported in epilepsy. Klassen *et al*. performed parallel exome sequencing of 237 ion channel genes in a well‐characterized human sample of epilepsy patients with presumed genetic origin (Klassen *et al*. 2011). Their study showed that disease risk from even deleterious ion channel mutations can be modified by variants in other ion channel proteins present in the same individual. In *CACNA1D* they found (in addition to coding synonymous, intronic mutations and one splice‐site mutation) one premature stop, and three missense mutations that occurred in probands but not in controls (Table 1). All of them occur in functionally sensitive regions of the channel. Two of them, A519P and Q1598X, are highly likely to affect channel function and have not been reported in > 120,000 alleles in the Exome Aggregation Consortium (ExAC) database. ExAC collects whole exome sequencing data from 60,706 unrelated individuals with no severe paediatric diseases as a useful reference set of allele frequencies for severe disease studies (Exome Aggregation Consortium (ExAC), Cambridge, MA, USA, http://exac.broadinstitute.org, accessed September 2015). A519P introduces a proline at the C‐terminal end of the cytoplasmic I–II linker. As recently shown this region forms a conserved polybasic helical structure that interacts with lipid headgroups at the plasma membrane. In Ca_v_1.2 channels it stabilizes channel function and mediates channel inhibition by phospholipase C‐induced phosphoinositide breakdown (Kaur *et al*. 2015). The high conservation of this motif suggests a similar role in all L‐type channels (Kaur *et al*. 2015). Like charge neutralizing mutations, the A519P mutation is therefore expected to interfere with normal channel gating and/or inhibitory control by receptor‐mediated phospholipid breakdown (Hille *et al*. 2014). Even more intriguing is variant Q1598X, truncating the C‐terminus of the Ca_v_1.3 α_1_ subunit. I1597 and Q1598 are part of the ‘IQ‐motif’ essential for calmodulin (CaM)‐mediated Ca^2+^‐induced inactivation of voltage gated Ca^2+^ channels, an important autoinhibitory mechanism preventing excessive Ca^2+^ influx (Van Petegem *et al*. 2005; Adams *et al*. 2014). Constitutively bound apo‐CaM enhances the Ca_v_1.3 open probability which is reversed upon Ca^2+^ binding (Adams *et al*. 2014). If the allele carrying mutation Q1598X results in the expression of a functional α_1_ subunit protein then apo‐CaM binding to this region must be decreased. This should reduce open probability but also Ca^2+^‐dependent inactivation making Ca^2+^ influx more long lasting. In the brain the function of this IQ‐motif is already heavily regulated by RNA editing (Huang *et al*. 2012) and alternative splicing (Shen *et al*. 2006; Tan *et al*. 2011) creating Ca_v_1.3 channels with reduced or absent CaM regulation. Mutant Q1598X channels could therefore enhance the number of channels with ‘CaM‐free’ gating properties, which may lead to enhanced Ca^2+^ signalling through persistent Ca_v_1.3 current components (Huang *et al*. 2012). Currently, a contribution of these mutations to overall epilepsy risk in the affected patients remains speculative. However, if gating changes compatible with gain of channel function can be demonstrated in heterologous expression studies of these mutants, this would strongly support a role for *CACNA1D* in idiopathic epilepsy. As discussed below, this has potential clinical implications since L‐type Ca^2+^ channel blockers available for treatment of hypertension could be beneficial in patients identified with such mutations.

**Table 1:** Missense mutations reported in *CACNA1D* in a patient cohort with idiopathic epilepsy (Klassen *et al*. 2011)

Missense			Potential pathogenic
mutations	Location in Ca_v_1.3 α_1_	ExAC variant	relevance for CNS disorders
A519P	End of cytoplasmic I‐II linker: CRA**A/P**VK	Not reported; only variant introducing a Thr rather than a Pro is described: 3:53756390 G/A (1 of 121408 alleles reported)	High: located close to previously reported regulatory polybasic plasma membrane binding motif (Kaur *et al*. 2015)
P1318S	Cassette exon in IVS3‐S4 linker present in some transcripts: ENV**P/S**VPT	Variant: 3:53804021 C/T (584 of 121400 alleles)	Unlikely: 4 homozygote carriers in ExAC; affects only specific splice variants
I1759N	Ile1761Asn in C‐terminus: RPS**I/N**GNL	Variant: 3:53835365 T/A (1 of 121318 alleles); (Ile→Leu and Ile→Met also reported in 1 allele each)	Unknown: mutation positioned one residue after proposed PKA phosphorylation site
Q1598X	Stop after Ile1597 of ‘IQ’ domain, essential for calmodulin binding; expected to affect 80% of *CACNA1D* transcripts; 20% do not contain the mutated residue	No variant reported	High: mutation removes C‐terminus including regulatory site for calmodulin; may give rise to more persistent Ca_v_1.3 currents

Only mutations observed in patients but not in matched controls are listed. Reference sequence is EU363339, except for P1318S (NM_000720). Amino acid sequence is given in single letter amino acid code; the position of mutated residues is indicated in bold.

## *CACNA1D* mutations associated with autism spectrum disorders

**Figure 2:**
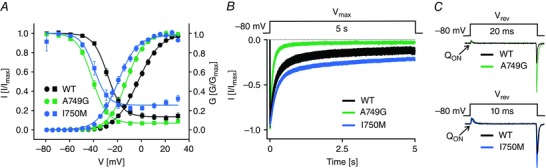
**Gating changes induced by missense mutations A749G (ASD) *vs*. I750M (PASNA, APA)** Mutations were introduced into the long isoform of Ca_v_1.3 α_1_ subunits and heterologously expressed in tsA‐201 cells together with auxiliary α_2_δ‐1 and β_3_ subunits. The biophysical characterization of the mutants was performed by whole‐cell patch‐clamp recordings with 15 mm Ca^2+^ as charge carrier. *A*, steady state activation (circles) and inactivation (squares). Data shown as the mean ± SEM. Mutation A749G (*n* = 27) shifted voltage dependence of activation by 10 mV to more negative potentials in comparison to wild‐type (WT, *n* = 29). Mutation I750M (*n* = 11) resulted in an even stronger change (−15 mV shift compared to WT). In addition, A749G (*n* = 14) shifted steady state inactivation (measured after 5 s conditioning pulses to the indicated voltages) by 15 mV to more negative potential compared to WT (*n* = 18). This shift was less pronounced for I750M (−8 mV, *n* = 9). *B*, *I_Ca_* inactivation upon 5 s depolarization to *V*_max_. Traces shown as the mean ± SEM. In contrast to I750M (*n* = 9) mutation A749G (*n* = 6) showed a more pronounced inactivation compared to WT (*n* = 15). The reduced inactivation of I750M is also evident in *A*. *C*, representative *I_Ca_* traces upon depolarization to the reversal potential (*V*_rev_) normalized to the area of the ON‐gating charge movement (*Q*_ON_, as a measure of functional channels on the cell surface). Mutations A749G (upper panel, green) and I750M (lower panel blue) showed increased tail amplitudes when normalized to *Q*_ON_ in comparison to WT (black), suggesting higher channel open probability or conductance. Modified from Azizan *et al*. (2013), I750M, and Pinggera *et al*. (2015), WT and A749G).

Whole exome sequencing also revealed two *de novo* mutations in autistic children of the Simons Simplex Collection (Figs 1 and 2). Intriguingly, these mutations are localized in close proximity to PASNA and APA mutations, suggesting a high contribution to disease risk in these patients (Fig. 1). As discussed in recent reviews (Striessnig *et al*. 2014; Zamponi *et al*. 2015), this is further supported by the known functions of Ca_v_1.3 for synapse formation and neuronal excitability as well as the fact that very similar mutations in structurally and functionally highly related Ca_v_1.2 channels (*CACNA1C* gene; see above) can also cause autism (Splawski *et al*. 2004).

Patient 1 harbouring one copy of the A749G mutation (O'Roak *et al*. 2012) is a Caucasian female and was diagnosed with ASD at 94 months. Her full IQ score was 62, the verbal and non‐verbal IQ scores of 67 and 65, respectively. The patient carried another risk mutation in *KATNAL2* with unknown physiological function (O'Roak *et al*. 2012) (patient information was obtained from the Simons Simplex collection, https://sfari.org/resources/autism‐cohorts/simons‐simplex‐collection, with permission and with approval by the local institutional review board).

Patient 2 carrying the G407R mutation (Iossifov *et al*. 2012) is a Hispanic male and was diagnosed at an age of 15 years with ASD. He has a full IQ score of 83, verbal and non‐verbal IQ scores of 81 and 88, respectively. In addition, a synonymous mutation in *ADAMTSL1* was found in this patient (Iossifov *et al*. 2012). He was also diagnosed with a congenital heart problem at 1 month. Seizures, anxiety or depressive disorders and hyperaldosteronism were not reported in the patient records.

When heterologously expressed in tsA‐201 cells together with accessory β‐ and α_2_δ‐subunits, both mutations induced strong functional changes very similar to the two PASNA mutations. G407R closely resembled G403D (compare data in Azizan *et al*. 2013 *vs*. Pinggera *et al*. 2015) whereas A749G resulted in alterations comparable to I750M (Azizan *et al*. 2013; Pinggera *et al*. 2015; Fig. 2). Both shifted the voltage dependence of activation and inactivation to more hyperpolarized potentials. This enables mutant channels to activate at more negative membrane potentials thus representing a gain‐of‐function phenotype. The hyperpolarizing shift of the voltage dependence of inactivation on the other hand reduces the amount of active channels at physiological potentials. Especially in neurons with more depolarized membrane potentials (e.g. dopamine neurons in the substantia nigra) this could also reduce channel availability. In addition, A749G resulted in faster inactivation kinetics during depolarizing stimuli compared to I750M (Fig. 2). Faster inactivation in turn is expected to decrease Ca^2+^ influx through Ca_v_1.3 channels in neurons during repetitive firing and might also reduce upstate potentials. Due to its non‐inactivating component, mutation I750M results in an increased window current, which represents the voltage range where the channels can be activated but are not completely inactivated, thus again representing a gain of function. A749G on the contrary reduces the window current due to the strong hyperpolarizing shift of the voltage dependence of inactivation and the faster inactivation kinetics. However, in fast spiking neurons a more negative activation range is expected to enhance Ca^2+^ current through A749G channels. Therefore the net effect of these mutations on Ca_v_1.3 Ca^2+^ signalling may vary depending on the firing pattern and action potential shape of a neuron.

Using exome sequencing, De Rubeis *et al*. (2014) analysed rare coding variations in 3871 autism cases and 9937 ancestry‐matched or parental controls. Many of the identified risk variants encoded proteins implicated in synapse formation, transcriptional regulation and chromatin remodelling pathways. Amongst the critical synaptic components found to be mutated were also voltage‐gated ion channels, including missense variants in *CACNA1D* and two loss‐of‐function variants in *CACNA2D3*, which encodes the α_2_δ‐3 subunit (interacting with different L‐ and non‐L‐type Ca^2+^ channels; for review Dolphin, 2013). This study confirmed the *de novo* A749G and G407R risk mutations, but also identified an additional *de novo* mutation (V584I) and three other risk variants (Fig. 1) in a case‐control population. None of the newly identified mutations has been functionally characterized so far. A59V is located in an N‐terminal CaM interaction site (NSCaTE; Dick *et al*. 2008) and may thus affect the inactivation properties of the channel. This region is also highly conserved in Ca_v_1.2 channels and the variant is not reported as a variant in the ExAC database.

Mutations S1953L and R1997H in the C‐terminal tail have been reported in the ExAC database (three S1953L, one S1953P; one R1997H, two R1997C). Moreover these residues are not conserved between Ca_v_1.3 and Ca_v_1.2. Their potential to contribute to ASD risk is therefore less obvious than for G407R and A749G. This is also true for the *de novo* mutation V584I for which two variants were reported in ExAC sequencing data.

If strong gain‐of‐function mutations as observed in APAs confer a high risk for PASNA and ASD, carriers are expected to present with CNS symptoms earlier in life and would therefore less likely show up in the ExAC control population, whereas this may not be true for mutations contributing lower risk. We screened the ExAC data for the presence of APA mutations and found variants corresponding to four mutated *CACNA1D* loci affected in APAs. One variant resulted in a glycine insertion after position 402 (p.Leu402_Gly403insGly) in exon 8b. An additional variant was reported in position 652, but in contrast to the corresponding APA mutation S652L (Fig. 2), this variant leads to mutation to a tryptophan. Mutation L655P was identified only once. Another mutation, V728I, was identified in 41 of > 121,400 alleles. All carriers were heterozygous for the variants. The glycine insertion at position 403 in *CACNA1D* leads to a non‐conducting channel due to impaired coupling of voltage sensing to pore opening (Baig *et al*. 2011). Therefore p.Leu402_Gly403insGly must result in a loss of function, which is not expected to cause a phenotype in the heterozygous state (Platzer *et al*. 2000; Baig *et al*. 2011). Mutations V728I, S652L and L655P have not been characterized so far. However, the fact that they all occur in the ExAC database suggests that the mutations, in particular V728I, might confer less risk to severe paediatric disease, perhaps by inducing more moderate gating changes.

## Conclusion

Taken together we hypothesize that gain‐of‐function *CACNA1D* mutations differ with respect to their contribution to CNS disease risk. Mutations causing strong changes in channel gating, similar to those reported in PASNA and some of the APA mutations (e.g. G403R, V259D), may convey a very high disease risk. Abnormal neuronal Ca^2+^ signalling by these dysfunctional channels may not be easily compensated by variants in other genes (e.g. encoding ion channels; Klassen *et al*. 2011) and thus lead to disease symptoms in the vast majority of carriers. On the other side of the spectrum, APA mutations such as V728I may manifest as primary aldosteronism without CNS symptoms in humans when present in the germline. This would explain the high number of carriers in the ExAC database. In the brain such rare *CACNA1D* variants inducing more subtle changes in Ca_v_1.3 function may only be of disease relevance in a permissive genetic background.

To prove this hypothesis more patients need to be identified with either PASNA, ASD with or without seizures or primary aldosteronism and strongly gating modifying *de novo* germline mutations in *CACNA1D*. If this is the case and the corresponding mutations (like G407R, A749G) retain their sensitivity to brain‐permeant L‐type Ca^2+^ channel blockers (such as isradipine, nifedipine or nimodipine), experimental therapies with these drugs in affected patients would be justified. If a contribution of such mutations to epileptic symptoms (in idiopathic epilepsy or as co‐morbidity in PASNA or, perhaps, autism) can also be demonstrated, these drugs may even be added to antiepileptic therapy.

In the last few years next generation sequencing has provided us with exciting novel insight into the aggregate burden of genetic variants. We are only at the beginning of understanding the role of *de novo* mutations in voltage‐gated L‐type Ca^2+^ channels for sporadic cases of CNS disorders. To better comprehend how distinct *CACNA1D* mutations contribute to different human disorders, further functional analysis of more high risk Ca_v_1.3 mutations reported in affected patients is important. Mutations activating these channels may not only confer enhanced risk for neuropsychiatric disease, as described in this article, but also for neurodegenerative disorders, in particular Parkinson's disease. Recent work has provided strong evidence that L‐type channel mediated Ca^2+^ entry contributes to oxidative stress underlying the high vulnerability of substantia nigra neurons to cell death (Sulzer & Surmeier, 2013). Therefore studying these human mutations in mice must be the next step to understand their disease‐causing potential in humans.

## Additional information

### Competing interests

The authors declare no conflicts of interest.

### Author contributions

Both authors have approved the final version of the manuscript and agree to be accountable for all aspects of the work. All persons designated as authors qualify for authorship, and all those who qualify for authorship are listed.

### Funding

The authors  work is supported by the Austrian Science Fund (FWF F44020, W1101), the University of Innsbruck and the Tyrolean Government.
